# Elevated pre-treatment levels of high sensitivity C-reactive protein as a potential prognosticator in patients with colorectal cancer

**DOI:** 10.3892/etm.2013.1350

**Published:** 2013-10-16

**Authors:** MAOSONG LIN, JUNXING HUANG, JIAYI ZHU, HONGZHANG SHEN

**Affiliations:** 1 Department of Gastroenterology, Taizhou People’s Hospital, Taizhou, Jiangsu 225300, P.R. China; 2 Department of Oncology, Taizhou People’s Hospital, Taizhou, Jiangsu 225300, P.R. China

**Keywords:** C-reactive protein, colorectal cancer, prognosis

## Abstract

Elevated levels of C-reactive protein (CRP) have been described as a prognostic factor in various types of human malignancy. In the present study, the prognostic potency of CRP was validated for patients with colorectal cancer (CRC) in order to guide patient management and define high-risk populations for follow-up or for therapeutic purposes. The association between the high sensitivity-CRP (hs-CRP) levels of a total of 123 patients with CRC and their clinicopathological characteristics was explored. Subsequently, univariate and multivariate analyses were performed to investigate the survival impact of pre-treatment hs-CRP levels in this cohort study. Statistically significant correlations between the serum levels of hs-CRP and lymph node and distant metastasis (P<0.001 and P=0.012, respectively), vascular and perineural invasion (P<0.001 and P<0.001), grades (P=0.022) and clinical stages (P=0.001), but not age and gender (P=0.616 and 0.676, respectively), were found. The five-year survival rate of patients with elevated (>5.0 mg/l) hs-CRP levels was demonstrated to be significantly less than that of those in the normal group (≥5.0 mg/l) by applying the Kaplan-Meier method (13.3 versus 57.0%, log-rank test P<0.001). Furthermore, following identification as a prognostic factor through using univariate analysis, high levels of hs-CRP (P<0.001) were validated as an independent prognosticator in CRC in the present study through using multivariate analysis. Pre-treatment serum CRP levels were associated with advanced and progressed CRC patients, therefore these levels may serve as a potential prognostic marker for CRC patients.

## Introduction

Colorectal cancer (CRC) is the third most common type of malignant tumor in Western countries, with an estimated total of 143,000 cases in the United States in 2010 (1). At present, against the declining trend of incidence of gastric cancer, the prevalence of CRC has maintained an upward momentum in China, which is partly attributable to acquiring the Western lifestyle. With the development of the algorithm of surgery and pan-operation systemic therapies, particularly chemotherapy and certain targeted therapies, the overall prognosis of patients has improved. However, certain patients, either in the curative setting or the metastatic one, with relatively similar clinical features may have varied clinical courses and their prognoses are difficult to predict. The underlying mechanisms of this phenomenon remain largely unknown. Therefore, prognostic markers are required in order to help stratify patients according to their risk, enable follow-up schedules to be individualised and tailor eligibility criteria for trials of neoadjuvant and adjuvant therapies. Tumor stage, pathological grade, lymph node involvement and lymphovascular invasion are commonly used as known prognostic factors in the clinic (2–4). However, all of these are postoperative factors. In addition, though certain abnormal tumor-associated genetic molecules were identified as being able to predict the prognosis of the patients (5,6), their measurement is to a certain extent time-consuming and complex and frequently not integrated into clinical practice. Therefore, identifying pre-treatment prognostic factors, including a number of serum biomarkers, would offer the opportunity for more objective and reproducible measurement and risk stratification prior to surgery. Thus, due to the characteristics of simplicity, cost-effectiveness and availablility in daily practice and the association with cancers, the measurement of serum C-reactive protein (CRP) levels has gained increasing attention.

CRP, a typical systemic inflammation marker, was first discovered in the plasma of patients during the acute phase of pneumococal pneumonia (7). It is produced almost exclusively in hepatocytes in response to inflammatory cytokines, such as intereukin (IL)-1, tumor necrosis factor (TNF)-α and, in particular, IL-6, within a few hours following insults such as infection, trauma or cardiovascular diseases (8,9). In recent decades, mounting evidence has demonstrated that elevated CRP levels were associated with an increased risk of malignancy (10–13). Moreover, elevated levels of CRP have been described as a prognostic factor in various types of human malignancy, including ovarian, and gastroesophageal (14–17). However, the correlation between CRP levels and prognosis in patients with CRC remains to be clarified (18,19).

To the best of our knowledge, few systematic studies focusing on the clinical significance of CRP in Chinese CRC patients have been reported. Therefore, in the present study, the potential clinical and prognostic significance of pre-treatment high sensitivity-CRP (hs-CRP) levels in Chinese patients with CRC of all stages and histological subtypes has been comprehensively analyzed.

## Patients and methods

### Patients

From 2005 to 2008, a total of 150 CRC patients were initially enrolled in the present study, including a number of cases from a previous retrospective study that investigated the clinical significance of platelet count in CRC (20). Patients underwent either en bloc colorectomy or palliative resection, and further chemotherapy in the inoperable patients was studied retrospectively. None of the patients had received preoperative chemotherapy. The histological tumor subtype was determined according to the 1997 UICC classification and staging was based on the 2002 TNM classification. Patients with active concurrent infection or who took non-steroidal anti-inflammatory drugs were excluded from the present study. Patients were also excluded if their pre-treatment CRP levels were unavailable. Information on patient and tumor characteristics, such as age, gender, stage, presence of regional lymph node or distant metastases, histological grade and CRP value, was obtained from the databases of Taizhou People’s Hopital (Taizhou, China). Only 123 patients (70 males and 53 females met the required inclusion criteria). Follow-up information, including the cause of mortality, was ascertained through a review of clinical notes and direct or family contact.

The Ethics Committee of Taizhou People’s Hospital approved the study. Written informed consent was obtained from all of the patients according to the guidelines approved by the Institutional Research Board of the hospital.

### Assay of serum CRP

A sample of the peripheral venous blood of patients was withdrawn one day prior to treatment. The blood samples were temporarily stored at 4°C. Immediately after the blood was centrifuged, serum samples or the supernatant were frozen and stored at −80°C until use. Pre-treatment hs-CRP values were measured as part of the clinical routine using a BN ProSpec system (Siemens Healthcare Diagnostics, Germany) according to the manufacturer’s instructions. Normal serum levels were defined as ≥5 mg/l by the manufacturer’s instructions. When investigating the correlation between the levels of hs-CRP with clinicopathological characteristics, the pre-treatment hs-CRP values were classified into the CRP ≥5 mg/l and >5 mg/l groups, according to the suggestion of Saito *et al*(21)and Stein *et al*(22).

### Statistical analysis

Comparisons between the two groups, the high levels of the hs-CRP and normal levels groups, were performed using the t-test for quantitative variables, the χ^2^ test for categorical clinical variables, and the Fisher’s exact test when appropriate. Overall survival was measured from the date of diagnosis until the date the patient succumbed due to disease or of the final follow-up. Survival curves were obtained according to the Kaplan-Meier method. Comparison of the survival curves was carried out using the log-rank test. Variables that were significant at P<0.05 in the univariate analysis were also included in the multivariate analysis. Multivariate survival analysis of the group variables was performed using the Cox proportional hazard model. Mortalities up to the end of May 2013 were included in the analysis. To remove a variable from the model, the corresponding P-value had to be >0.10. Analysis was performed using SPSS software, version 19.0 (SPSS Inc., Chicago, IL, USA) and two-tailed values of P<0.05 were accepted as indicating a statistically significant difference.

## Results

### Patient characteristics

In total, 123 patients with CRC in this cohort (70 male and 53 female) met the inclusion criteria. The details of the baseline clinicopathological parameters of the patients studied are listed in Table I. The mean age of the group whose hs-CRP levels were >5 mg/l was 62.07±12.51 years, while that of the normal group was 60.85±11.21 years, which did not manifest a significant difference (P=0.616). A minor dominance of male cases (n=70) was observed compared with their female counterparts (n=53) overall, but no significant difference of gender distribution was identified between the two groups. In addition, most of the patients were classified as stage III, followed by stage II, according to the TNM classification, and only five patients with stage IV and two with stage I were included in this cohort study. In total, 56 patients, 25 in the hs-CRP >5 mg/l group and 31 in the hs-CRP <5 mg/l group, exhibited regional lymph node involvement. However, significantly more cases of lymph node-negative patients were identified in the hs-CRP levels group compared with the normal group (P<0.001). Partly attributable to the surgical indication, there were only five patients with distance metastasis which had palliative resection performed on them in this cohort of cases, of which four cases had significantly elevated levels of hs-CRP.

### Clinicopathological significance of hs-CRP

The cut-off point for measurements of serum hs-CRP levels was set at 5.0 mg/l in the hospital, which is concurrent with previous studies (21,22). When the patients were divided into two groups according to the individual baseline levels of hs-CRP, 30 patients (24.39%) had pre-treatment serum hs-CRP levels above the defined cut-off point of 5 mg/l, ranging from 5.02 to 56.54 mg/l. Conversely, no serum elevation of hs-CRP levels was recognized in the 93 patients (75.61%) who were assigned into the normal group (Table I). A close association was observed between elevated hs-CRP levels (CRP>5.0 mg/l) and clinical lymph nodal status (P<0.001), distant metastasis (P=0.012), vascular and perineural invasion (P<0.001 and P<0.001), tumor differentiation (P=0.022) and clinical stage (P=0.001) (Table I).

**Figure 1:**
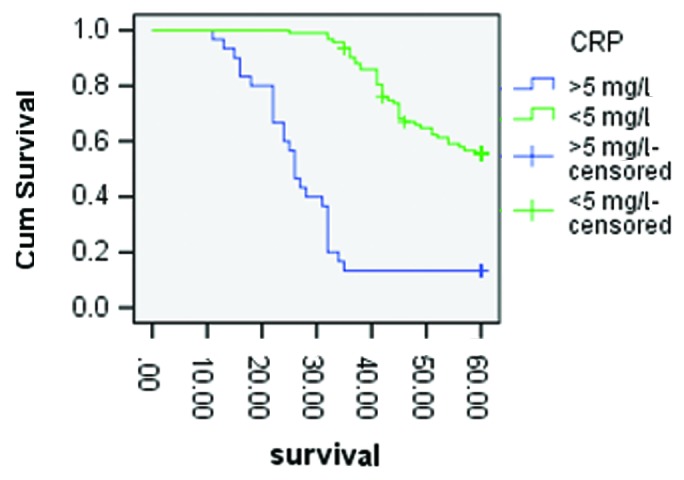
Survival of patients according to the pre-treatment hs-CRP levels. The five-year survival rate of patients with high serum levels of hs-CRP was 13.30%, in comparison to 57.0% of the normal group (P<0.001). hs-CRP, high sensitivity-C reactive protein.

The mean duration of follow-up for the survivors (n=57) was 35.5 months (range, 11–60 months). Overall, more patients in the high serum hs-CRP levels group had succumbed to disease (26/30) in comparison to their normal counterpart after five years of follow-up, of which 60 patients succumbed as a result of progression of the CRC, two due to surgical complications and four due to non-cancer causes (data not shown). As shown in Fig. 1, the five-year survival rates of the patients with high levels of hs-CRP and normal levels were 13.30 and 57.0%, respectively (P<0.001). Following the univariate analysis, along with the conventional prognostic factors such as lymph node and distant metastasis, vascular and perineural invasion, clinical stage and pathological grades, serum hs-CRP levels were also demonstrated to be correlated with the life span of the patients. Furthermore, considering results of the multivariate analysis, perineural invasion [hazard ratio (HR) 6.181, 95% confidence interval (CI) 1.264–30.241, P=0.025], clinical stage (HR 6.86, 95% CI 2.045–23.01, P=0.002) and hs-CRP >5 mg/l (HR 5.196, 95% CI 2.901–9.309, P<0.001) were also identified as independent prognosticators in CRC in this study (Table II).

## Discussion

In 1863, Virchow identified leukocyte infiltration in neoplastic tissues and suggested these sites of chronic inflammation were the origin of cancer, which was the first suggestion of the linkage between inflammation and cancer (23). Since then, epidemiological links between *Helicobacter pylori* bacterial infection and gastric cancer and mucosa-associated lymphoid tissue lymphoma, the so-called MALToma, as well as inflammatory bowel disease and CRC validated this hypothesis (24–26.). Notably, CRP, a systemic inflammation marker, was reintroduced as a tool for monitoring malignancies, similar to its use in cardiovascular diseases in recent decades (9,27,28). Furthermore, elevated levels of CRP have also been described as a prognostic factor in various types of human malignancy, including digestive system cancers (14–16,29,30). The majority of the previous studies presumed that elevated serum CRP levels in patients with malignancy were probably a bodily response, secondary to tumor necrosis, local tissue damage and associated inflammation through the cytokines released from leukocytes infiltrating within the tumor microenvironment, in particular IL-6 (31).

To the best of our knowledge, several studies have explored the pathological role of CRP in CRC in the Western countries. Of the nine published retrospective studies up to 2011, three studies demonstrated positive associations between circulating CRP levels and CRC incidence (32–34), while the remaining reports indicated generally null (35–38) or even inverse (39,40) associations, which purported the discrepant conclusions presented. However, two individual meta-analyses which summarized data from 8 and 11 independent prospective investigations, respectively, concluded that CRP was a weak, positive risk factor for CRC (41,42). However, few studies are currently available with regard to this issue in Chinese CRC patients. Therefore, in the present study, the clinical significance of hs-CRP was first explored in CRC patients in the region. In the cohort of cases, 30 patients with high serum hs-CRP levels according to our cut-off line set as >5 mg/l were identified. At the same time, no significant association of the hs-CRP levels with regard to the age and gender of the patients was noted. However, it is encouraging that hs-CRP levels were notably increased in the cases with lymph node or distant metastasis and vascular or perineural invasion, all of which are traditional pathological prognostic factors. Elevated hs-CRP concentrations in serum were more common in the patients with advanced stage CRC. These results suggest the predictable potency of CRP in the mortality of CRC patients. Therefore, the results of the present study to a certain extent confirmed the conclusions of a number of previous studies which considered the positive pathological role of CRP in CRC (32–34,43). Possible reasons for the difference between the previous studies which indicated a negative association between CRP levels and clinical and pathological features in CRC patients, and other previous positive studies including the present one are probably partly attributable to the variable potential biological features, different stages, distribution of the patient population and the disparate territory.

Conventional factors, such as pathological clinical stages and lymph node involvement, are the most important predictors of poor clinical outcome in clinical practice (44–46). Furthermore, a number of less common serological and molecular markers have also shown prognostic evidence, such as the estrogen receptor, progesterone receptor, human epidermal growth factor receptor-2 in breast cancer and mismatch repair gene in stage II CRC (6,7). However, these markers are only available following surgery and their measurement is, to a certain extent, time-consuming and complex and therefore are often not integrated into clinical practice. By contrast, serum CRP levels, measurement of which is relatively inexpensive and easy to quantify in daily clinical practice, allows classification of patients who are at a relatively high risk and who are candidates for the latest intensive treatment. In the last 10 years, an increasing amount of evidence suggesting the CRP prognostic role in various types of tumors, including CRC, has been reported (47,48). Similarly, in the present study, whether CRP had a prognostic role in this cohort of patients was also investigated. Notably, following a further univariate analysis, concurrent with lymph node or distant metastasis, vascular or perineural invasion and clinical stage, elevated hs-CRP levels were identified as a prognostic marker in CRC. Patients with high serum levels of hs-CRP had relatively poorer five-year survival rates in comparison to their normal hs-CRP counterparts, suggesting that high concentrations of hs-CRP are a potential prognostic determinant. In addition, when included in the multivariate analysis, hs-CRP was also indicated as an independent prognostic factor in the cohort of cases. Together with perineural invasion, clinical stage was confirmed as another prognostic factor in the present study. However, although HR was manifested as >1, the prognostic significance of lymph node and distant metastasis were not indicated in this study, which was not in accordance with previous results (19,20). Relatively fewer patients with distance metastasis recruited in the present study may have contributed to the contrary result. Overall, the CRC patients with high levels of pretreatment CRP may demonstrate high risk potential, thus more attention should be given this issue in the consideration for more active therapies. Despite the significant results obtained in this study, it should be noted that this is a relatively small study in a single center and further verification in large cohorts in multiple centers in China is required.

To investigate the potency of CRP in CRC, it is essential to define CRP, i.e., a participant in the pathogenesis of CRC or simply a marker of CRC. However, the role of CRP in cancers, including CRC, remains poorly understood. Several mechanisms of increased CRP levels in malignant tumors are now known due to various therories. First, serum CRP levels may reflect the aggressiveness of the tumor, as they are the result of the immune response of the host to tumor growth (49,50). Second, tumor growth causes tissue inflammation in the tumor microenvironment by increasing the production of inflammatory proteins, particularly IL-6. Immune and inflammatory cells in the tumor microenvironment interact with malignant cells in a complicated manner and the net result of which is stimulation of tumor growth, invasion and metastasis (51–53). As a result, the exact underlying role of CRP in various types of cancer including CRC requires more biological exploratory studies in the future.

In conclusion, the pre-treatment serum CRP levels may be a marker of aggressive characteristics of in Chinese CRC patients. Elevated CRP levels prior to initial treatment were demonstrated to be a poor prognostic factor for the overall survival of CRC patients in China. Due to its increased attractiveness as a routinely available, relatively inexpensive and objectively measured marker available pre-operatively, CRP may complement the prognostic value of traditional prognostic factors, such as stage and performance status, to more accurately stratify patients with CRC. However, the results of the present study should await internal or external validation in a number of centers and prospective exploratory studies prior to being used in clinical practice in China due to the limitations inherent to a retrospective study with a small sample size.

## Figures and Tables

**Table I:** Association between pretreatment hs-CRP levels and baseline clinicopathological variables in patients with CRC.

	Groups		
		
Variables (n)	hs-CRP ≥5 mg/l	hs-CRP >5 mg/l	P-value
Total no. of cases	93	30	
Age (year; mean ± SD)	60.85±11.21	62.07±12.51	0.616
Gender			
Male	54	16	0.676
Female	39	14	
Lymph node invasion			
Positive	31	25	<0.001
Negative	62	5	
Distant metastasis			
Positive	1	4	0.012
Negative	92	26	
Vascular invasion			
Positive	8	15	<0.001
Negative	85	15	
Perineural invasion			
Positive	8	17	<0.001
Negative	85	13	
Histological grade			
Low	39	17	0.022
Moderate	37	13	
High	17	0	
TNM stage			
I	2	0	0.001
II	24	1	
III	66	25	
IV	1	4	

hs-CRP, high sensitivity-C reactive protein; CRC, colorectal cancer.

**Table II:** Univariate and multivariate analysis of the clinicopathological parameters in CRC.

	Overall survival				
	
	Univariate analysis		Multivariate analysis		
		
			95.0% CI		
				
Variables	P-value	Hazard ratio	Lower	Upper	P-value
Age	0.621				
Gender	0.544				
hs-CRP >5 mg/l	<0.001	5.196	2.901	9.309	<0.001
Lymph node invasion	<0.001	1.576	0.875	2.837	0.129
Distant metastasis	<0.001	1.146	0.223	5.878	0.87
Vascular invasion	<0.001	0.585	0.126	2.724	0.494
Perineural invasion	<0.001	6.181	1.264	30.241	0.025
Histological grades	0.003	0.972	0.63	1.499	0.897
TNM stages	<0.001	6.86	2.045	23.01	0.002

CRC, colorectal cancer; CI, confidence interval; hs-CRP, high sensitivity C-reactive protein.
